# The effect of gender stereotypes on young girls’ intuitive number sense

**DOI:** 10.1371/journal.pone.0258886

**Published:** 2021-10-28

**Authors:** Antonya Marie Gonzalez, Darko Odic, Toni Schmader, Katharina Block, Andrew Scott Baron

**Affiliations:** 1 Department of Psychology, Western Washington University, Bellingham, Washington, United States of America; 2 Department of Psychology, University of British Columbia, Vancouver, British Columbia, Canada; 3 Department of Psychology, New York University, New York, New York, United States of America; French National Center for Scientific Research (CNRS) & University of Lyon, FRANCE

## Abstract

Despite the global importance of science, engineering, and math-related fields, women are consistently underrepresented in these areas. One source of this disparity is likely the prevalence of gender stereotypes that constrain girls’ and women’s math performance and interest. The current research explores the developmental roots of these effects by examining the impact of stereotypes on young girls’ intuitive number sense, a universal skill that predicts later math ability. Across four studies, 762 children ages 3–6 were presented with a task measuring their Approximate Number System accuracy. Instructions given before the task varied by condition. In the two control conditions, the task was described to children either as a game or a test of eyesight ability. In the experimental condition, the task was described as a test of math ability and that researchers were interested in whether boys or girls were better at math and counting. Separately, we measured children’s explicit beliefs about math and gender. Results conducted on the combined dataset indicated that while only a small number of girls in the sample had stereotypes associating math with boys, these girls performed significantly worse on a test of Approximate Number System accuracy when it was framed as a math test rather than a game or an eyesight test. These results provide novel evidence that for young girls who do endorse stereotypes about math and gender, contextual activation of these stereotypes may impair their intuitive number sense, potentially affecting their acquisition of formal mathematics concepts and developing interest in math-related fields.

## Introduction

Women continue to be highly underrepresented in mathematics, engineering, and related fields; a pattern that is associated with cultural stereotypes associating math more with men than women [1–3]. A large body of correlational and experimental work has linked these gender stereotypes to a gender gap in math performance and has suggested that subtle reminders of gender stereotypes can sometimes cause some women to underperform on tests of their math ability [4–7]. Because gender stereotypes can emerge in elementary school, research also suggests that they can impair school-aged girls’ math performance when those stereotypes are contextually activated [8–12]. However, some scholars have also questioned the strength of evidence for the performance impairing effects of stereotype activation, especially among children [13, 14]. The present research examined 3 to 6-year-old childrens’ beliefs about gender and math, as well as the relationship between these beliefs and early math ability. Specifically, we examined whether especially young girls who endorse cultural stereotypes associating math with boys early in development, might be susceptible to gender cues impairing math ability even *before* they enter formal education (see [15] for a review).

There are three key reasons why there might be variability in the degree to which young girls endorse stereotypes about girls and math. Firstly, given evidence that many stereotypes and biases have been weakening over time [16, 17], there is likely variation in the degree to which children hold the stereotype that girls are inferior in math. Alternatively, these stereotypes may be precluded by a strong in-group preference, as many children show preference for their own gender as early as age 3, which may lead them to believe their own gender is better at math [18]. Lastly, children may internalize these stereotypes at different points in development (e.g. [19, 20]), with some children internalizing cultural stereotypes about math as early as age 5, and others internalizing these stereotypes toward the end of elementary school (ages 9–10).

Though there are several reasons to expect that not all young children will have the same knowledge or beliefs about gender stereotypes regarding math ability, prior research on the effect of math-gender stereotypes on children’s math performance has not conventionally measured individual variability in children’s knowledge or endorsement of stereotypes [13, 14], despite the fact that knowledge of the stereotype is a core assumption of stereotype threat theory. Thus, to understand the effect of situational stereotype activation on children’s math performance, it is essential for researchers to measure whether children have internalized these stereotypes in the first place. In adults, for example, women who have not internalized stereotypes about math and gender seem to be less affected by a manipulation intended to impair their math performance through stereotype activation [21]. Additionally, in some cultural contexts, the activation of gender stereotypes might even have reverse effects such that a mention of gender differences in math can lead boys to underperform relative to girls [22].

In addition to lacking measurement of children’s existing stereotypes about gender and math, the current body of research on stereotype-based performance impairments has typically examined effects on formal math tests. Using formal math tests can limit our understanding of how these stereotypes affect math ability, as children acquire the skills tested on these assessments through a combination of individual interest and educational experience typically starting around age five or six. Present from birth (or shortly after), children (and many non-human animals) have a more basic, universal, and intuitive number sense often termed the Approximate Number System (ANS) [see [23, 24] for reviews]. The ANS provides us with our gut-based sense of number (e.g., in our ability to quickly but approximately estimate the number of items in a visual display). Among humans, this capacity appears to be predictive of the later acquisition of formal, symbolic math abilities. Interestingly, there is some variation in the acuity of ANS representations within cohorts. Children and adults who have a very precise number sense perform substantially better on various formal and informal math assessments, even when controlling for working memory, intelligence, and other related variables [24–28]. Importantly, this system can also be modulated: adults and children (ages 5–7) who have their ANS temporarily boosted through training or feedback perform better on a subsequent math test, and when ANS acuity is reduced through these methods, they perform worse [29–31].

The current research uses ANS performance as a measure of math ability to examine the relationship between children’s beliefs about math and gender and their math ability prior to extensive exposure to formal mathematics education. Critically, despite a lack of overall sex differences in ANS capabilities [32], we explore whether contextual activation of gender stereotypes might impair the ANS accuracy of girls who have internalized the belief that boys are inherently better at numbers and math. As this system helps with the acquisition of formal mathematics skills, any stereotype-based impairments of the ANS that operate in early childhood, before formal math education, would only compound in degree over time, potentially impairing girls’ acquisition of formal mathematics concepts and their developing interest in math-related fields. Thus, an understanding of how stereotypes affect girls’ more basic numerical cognition is crucial to ensure that girls and boys do not begin their formal math education on unequal footing.

In the present research, we ran four studies examining the relationship between children’s beliefs about gender and math and their ANS performance in situations that cue gender stereotypes. We tested the hypothesis that 3–6 year-old girls who have already internalized gender stereotypes about counting and math would exhibit impaired ANS accuracy when the task is described as a measure of math and counting, but not when the task is described as a game (Study 1 & 2) or an eye test (Study 3 & 4). To our knowledge, this is the first research to assess these questions in children at this early age. As will become clear, results suggested that task description can impair girls’ ANS accuracy but only for those who have already internalized the gender stereotype favoring boys as superior at math. However, because this subset of girls is relatively small (making up approximately 12% of girls in the total sample; n = 60), we sought to maximize statistical power by analyzing data on a combined sample of these four datasets using a mega-analytic approach. This combined analytic approach was pregistered prior to conducting Study 4.

## Materials and methods

### Open practices statement

Our study protocol received ethics approval from the University of British Columbia Behavioural Research Ethics Board, #H10-0047. Written parental consent was obtained for all participants. Methods and analyses for Study 4 were preregistered. Additionally, we preregistered combining this study with previous studies to increase the power of our analysis. The preregistration for the current research can be found at https://osf.io/va7gs. The combined dataset and analysis scripts can be found at https://osf.io/yg4be. Supporting Information contains additional results and tables.

### Participants

We tested a total of 762 children (498 girls, 264 boys) ages 3–6 across four samples (see S1 Fig). Though our main hypotheses focus on girls, in Study 1–3 we also collected data from boys as comparison to test the specificity of effects. Data collection took place at a community-based science center. An additional 283 children were tested but excluded from analyses for pressing the buttons randomly or in a fixed pattern, failing to finish the study, parent or sibling interference, language barriers, any computer or experimenter error, disclosed neurodivergence, or scoring below chance levels on the ANS task (< 50% of trials correct; S1 Fig). These exclusion rates are typical of community testing environments, where a higher proportion of children are excluded as compared to traditional university lab settings (see [33]). Our *a priori* goal was to run 60 useable children per gender and age group (3–4 year olds, who have not begun formal schooling and 5–6 year olds, who have entered kindergarten) in each study (i.e., n = 240 per study), and we stopped running participants after we believed we had met this goal. Participants were recruited by research assistants who approached potential families at the community-based science center, reviewed the study description, and sought parental consent and child assent to participate. Children were tested onsite in an area dedicated for behavioral science research.

### Procedure

Parents were first asked to consent to the study, and then reported their child’s date of birth and gender identity to research assistants. After consent procedures, and after obtaining verbal child assent to participate, participants were tested individually in a soundproof room dedicated to behavioral science research. The experiment was presented on a computer using Inquisit™ version 4, and an experimenter read all instructions aloud to children. We quasi-randomly assigned children to condition by alternating which condition they were in but balancing this assignment across age and gender.

In all studies, children were presented with instructions before the ANS task based on condition. In each study, one condition was intended as a control condition, and the other was intended to prime gender stereotypes about math. Study 1 and 2 had two conditions: the game control condition and the math test condition. In the game control condition, children were given the following instructions: “Now we’re going to play a game. Your job is to try your best”. In the experimental (math test) condition, children were given the following instructions: “Now we’re going to test your math ability. This test tells us whether boys or girls are naturally better at math and counting.” In Study 3 and 4, the math test condition was identical to Study 1 and 2. However, to further control for priming of gender and possible effects of simply calling the task a ‘test,’ we modified the wording of our control condition. Specifically, in our control condition for these studies, children were told: “Now we’re going to test your eyesight ability. This test tells us whether boys or girls are naturally better at seeing things quickly.”

Afterwards, all children were presented with the ANS task and given the same task instructions (see Approximate Number System Task below). In Study 1, 2, and 4, after the ANS task, children were presented with stereotype belief questions. In Study 3, the order of presentation for the ANS task and the stereotypes were counterbalanced, with half of participants completing the ANS task first, and the other half of participants answering the questions first (order did not affect results; see Supplemental Online Materials). Upon completion of the study, all children were given a sticker for participation, and parents were debriefed on the aims of the research.

### Measures

#### Approximate Number System (ANS) task

We measured each child’s ANS accuracy using the standardized Panamath test [27]. Participants were introduced to Big Bird and Grover–two characters drawn on the screen, each of whom had an empty box that was color matched to their character (yellow and blue respectively). Participants were told to decide which character had more dots in their box on each trial. For participants ages four and above, children pressed a corresponding yellow and blue JellyBean™ button based on which character they thought had more dots. Participants who were three years old were simply asked to point to the character they thought had more dots, and the experimenter would answer for them using the keyboard.

**Fig 1:**
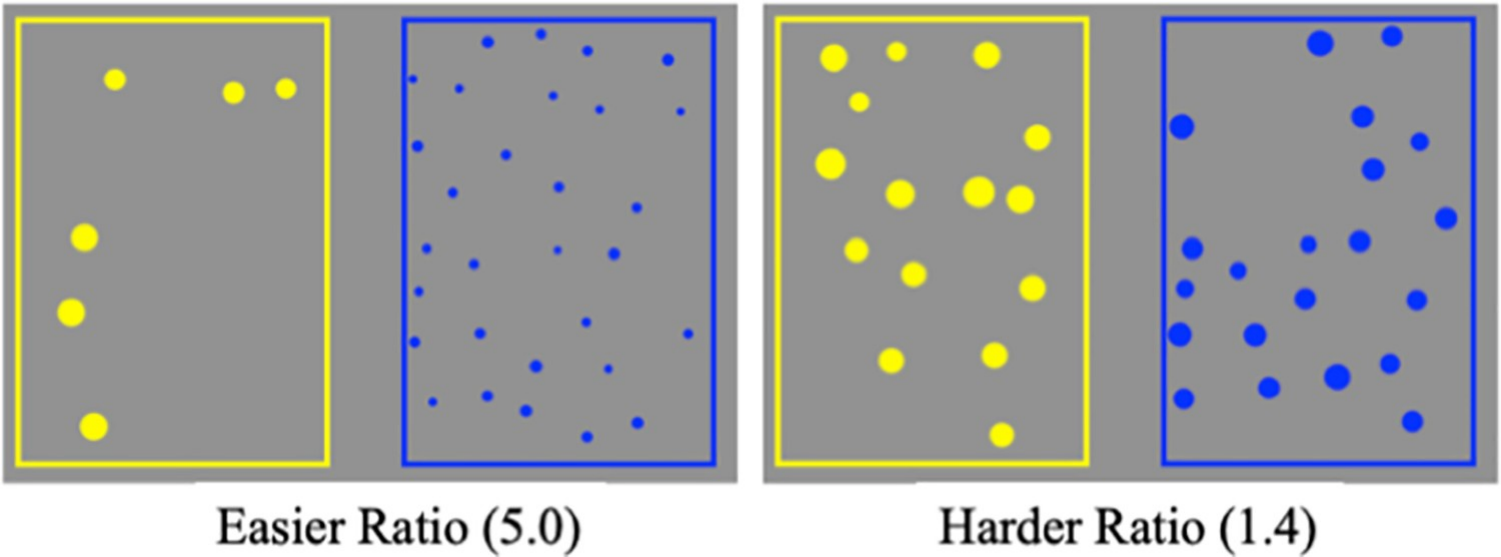
Approximate Number System (ANS) trial examples.

For each trial, two arrays of colored dots (yellow and blue) appeared in their respective boxes for 1500 milliseconds (Fig 1). To control for the difficulty of the task, children were presented with different numerical ratios based on published norms for their age. In Study 1, we used these pre-programmed ratios in the Panamath software [27]. In Study 2–4, ratios were more accurately customized for age norms [34]. Half of trials had a cumulative surface area that was congruent with the number of dots, and on the other half of trials, this was incongruent, which controls for the possibility that children might be conflating judgments of number with judgments of area. Children wore headphones during the task and received either positive or negative verbal feedback from the program based on performance on each trial, but were not able to correct their previous mistakes. All children included in our final sample completed 80 trials.

After completing the task, children in the control conditions were told: “Great job! We’ve found that boys and girls both really like playing that game.” In the test conditions, children were told: “Great job! We’ve found that boys and girls do equally well on that test.” We added these statements as a debriefing measure to ensure that children did not make later judgments based on their own performance (e.g., generalizing from their own experience of their performance to how others of the same gender identity may perform).

#### Math-gender stereotypes

Children were presented with four questions about their math-gender beliefs, specifically, two questions about math ability and two questions about math interest [8]. For ability questions, experimenters would ask: “Which person do you think is better at math and counting? Do you think this person (on the left) is better at math and counting, this person (on the right) is better at math and counting, or are they the same?” For interest questions, experimenters would ask: “Which person do you think likes math and counting more? Do you think this person (on the left) likes math and counting more, this person (on the right) likes math and counting more, or do they like it the same?” Questions about ability (“Which child is better at…”) and interest (“Which child likes…”) were always blocked together, but the order of these blocks was counterbalanced across participants.

For all questions an image of a cartoon boy and girl was presented on the computer screen. Furthermore, the ethnicity and skin tone of the cartoon children varied across trials. The boy and the girl in each trial were always the same race.

For each trial, children could indicate the boy, the girl, or indicate that they thought the boy and the girl were the same by either pointing or verbalizing their response. For purposes of interpretation, we coded these responses in relation to participants’ own gender (0 = other gender is better at math, 1 = no gender bias, 2 = own gender is better at math), referred to as math-own gender beliefs in our analyses.

#### Control stereotype measure

In Study 2, in addition to measuring math-gender stereotypes, we included control trials to ensure that children were not simply selecting one gender regardless of question content. Children were presented again with images of a boy and a girl on the same screen and asked two questions in the same style as the stereotype measures. First, they were asked which of the two children was better at “daxing” and then which of the two children liked “daxing” more. Some children received questions about math-gender stereotypes first, and others received questions about daxing first. See Supporting Information for all analyses related to this measure (S2 Text).

## Results

### Analytic approach

To maximize statistical power for testing the predicted three-way interaction (math-gender beliefs x child gender x condition), we present the results conducted on the combined dataset created by aggregating the four studies (N = 762; see Open Practices statement above). According to a sensitivity analysis conducted using G*POWER, this sample size would give us 95% power to detect significant regression coefficient in our model with a small effect size (f^2^ = 0.017) [35]. After conducting Studies 1–3, we preregistered hypotheses for Study 4 as well as the intention to analyze the four study combined dataset. This mega-analytic approach is generally preferable to meta-analysis (i.e., estimating the true effect size from sample-level effects), when the raw data are available [36–38]. It also in line with a growing preference for fewer well-powered studies [39], and recommendations to pool multiple small samples to boost power when testing higher order interactions and to provide more stable estimates of effect sizes [40]. Results for each individual experiment are summarized under Individual Study Results and detailed in the Supplemental Online Materials. While not all effects are identical across the four studies, this variation is to be expected within multi-study data (see [41, 42]).

### Math-gender beliefs

Our first set of analyses examined children’s beliefs about math and gender in our combined sample, which were coded such that a higher score would indicate a stronger association between children’s own gender and math (Table 1). A one-sample t-test evaluating children’s math-own gender beliefs against chance (midpoint = 1, indicating no preference), indicated that, on average, children associated math with their own gender (*M* = 1.20, *SD* = 0.42; *t*(761) = 13.28, *p* < .001), which is consistent with prior literature and underscores the importance of examining the moderating role of these beliefs. We found no gender difference in the magnitude of math-own gender beliefs, as an independent samples t-test indicated boys (*M* = 1.20, SD = 0.47) and girls (*M* = 1.20, SD = 0.39) had comparable average associations between their own gender and math, *t*(459.25) = .091, *p* = .93, *d* < 0.001 (t-test uses corrected values due to unequal variance, *p* = .001). Furthermore, a one-sample t-test evaluating children’s math-own gender beliefs against chance (midpoint = 1, indicating no preference), revealed that both boys and girls on average associated their own gender with math, boys: *t*(263) = 6.91, *p* < .001; girls: *t*(497) = 11.58, *p* < .001. There was no difference in the magnitude of math-own gender beliefs across conditions, as an independent samples t-test indicated that mean levels were comparable across the combined control and math test conditions, *t*(760) = 0.37, *p* = .71, *d* < 0.001. Lastly, a Pearson’s product-moment correlation indicated that math-own gender beliefs were not significantly correlated with age for girls, *r* = -.02, *p* = .66 or boys, *r* = .11, *p* = .06, suggesting that beliefs about math and gender were not changing significantly across the age range of our sample.

**Table 1:** Frequencies and mean age by gender, condition, and math-gender beliefs.

	Girls		Boys		All	
Variable	N	Age	N	Age	N	Age
Condition						
Control	245(49.2%)^a^	5.16(0.96)^b^	131(49.6%)	4.94(1.01)	376(49.3%)	5.08(0.98)
Math	253(50.8%)	5.11(1.05)	133(50.4%)	5.00(0.97)	386(50.7%)	5.07(1.02)
Total	498	5.13(1.00)	264	4.97(0.99)	762	5.07(1.00)

^a^Values in parentheses indicate percentage of participants of each gender.

^b^Values in parentheses indicate standard deviation.

### ANS task performance

Our second set of analyses concerned overall ANS performance and potential age and gender differences on this measure. ANS performance was quantified as children’s overall accuracy across the 80 trials of the task. Across all studies children performed well: on average they correctly answered 80.61% of trials (boys: 77.44%; girls; 82.29%). Consistent with other work on children’s ANS, a Pearson’s product-moment correlation indicated task accuracy increased with age, *r* = .31, *p* < .001 (boys: *r* = .24, *p* < .001; girls: *r* = .34, *p*s < .001). Using an independent samples t-test, we also found an overall gender difference counter to gender stereotypes, with girls performing better on the task than boys, *t*(507.80) = -5.59, *p* < .001, *d* = .43 (t-test uses corrected degrees of freedom due to unequal variance, *p =* .03).

### Predictors of ANS accuracy

Our third and key set of analyses tested the hypothesis that in the math test condition, as a result of making math-gender beliefs relevant to the task, a stronger association between one’s own gender and math would predict better ANS performance. We expected no such relation in the combined control condition. Further, we tested child gender as a potential moderator. To test this hypothesis, we performed a series of stepwise regression analyses (see Table 2), first controlling for sample by creating three dummy coded variables to represent the four different studies and entering these variables as predictors of ANS performance in Step 1. We then entered math-own gender beliefs (standardized), child gender (dummy coded; 0 = female, 1 = male), and condition (dummy coded; 0 = control, 1 = math test) in Step 2. We then entered two-way interactions between gender and condition, condition and math-own gender beliefs, and gender and math-own gender beliefs as additional predictors in Step 3. Finally, in Step 4, we entered a three-way interaction between gender, condition, and math-own gender beliefs. Follow-up analyses including age as a possible moderator in the model yielded no significant main effects or interactions by age.

**Table 2:** Gender and math-own gender beliefs predicting ANS accuracy.

Predictor	β	*SE*	*p*	Δ*R*^*2*^	Model Sig.
Step 1				-.06	< .001
Study 1 (Study 2,3,4 = 0; Study 1 = 1)	-0.61	0.12	< .001^a^		
Study 2 (Study 1,3,4 = 0; Study 2 = 1)	-0.26	0.09	.006^a^		
Study 3 (Study 1,2,4 = 0; Study 3 = 1)	-0.53	0.09	< .001^a^		
Step 2				-.02	< .001
Study 1 (Study 2,3,4 = 0; Study 1 = 1)	-0.45	0.13	< .001^a^		
Study 2 (Study 1,3,4 = 0; Study 2 = 1)	-0.12	0.10	.23		
Study 3 (Study 1,2,4 = 0; Study 3 = 1)	-0.39	0.10	< .001^a^		
Gender (F = 0; M = 1)	-0.28	0.08	< .001^a^		
Condition (Control = 0, Math = 1)	-0.08	0.07	.22		
Math-Own Gender Beliefs	0.001	0.03	.97		
Step 3				-.008	< .001
Study 1 (Study 2,3,4 = 0; Study 1 = 1)	-0.44	0.13	< .001^a^		
Study 2 (Study 1,3,4 = 0; Study 2 = 1)	-0.12	0.10	.23		
Study 3 (Study 1,2,4 = 0; Study 3 = 1)	-0.38	0.10	< .001^a^		
Gender (F = 0; M = 1)	-0.34	0.11	.002^a^		
Condition (Control = 0, Math = 1)	-0.12	0.09	.18		
Math-Own Gender Beliefs	-0.05	0.06	.39		
Gender x Condition	0.10	0.15	.50		
Gender x Beliefs	-0.06	0.07	.36		
Beliefs x Condition	0.16	0.07	.022^a^		
Step 4				-.006	< .001
Study 1 (Study 2,3,4 = 0; Study 1 = 1)	-0.43	0.13	< .001^a^		
Study 2 (Study 1,3,4 = 0; Study 2 = 1)	-0.13	0.10	.21		
Study 3 (Study 1,2,4 = 0; Study 3 = 1)	-0.37	0.10	< .001^a^		
Gender (F = 0; M = 1)	-0.34	0.11	.002^a^		
Condition (Control = 0, Math = 1)	-0.12	0.09	.17		
Math-Own Gender Beliefs	-0.12	0.07	.07		
Gender x Condition	0.10	0.15	.49		
Gender x Beliefs	0.09	0.10	.37		
Beliefs x Condition	0.30	0.09	.001^a^		
Gender x Beliefs x Condition	-0.32	0.14	.023^a^		

^a^Statistically significant regression coefficients at an alpha = .05.

#### Experiment predicting ANS performance

Results of this analysis revealed that experiment was a significant predictor of ANS performance, which appeared to be primarily driven by Study 4 (*M* = 84.06%) where participants outperformed the participants in Study 1 (*M* = 77.10%), Study 2 (*M* = 81.12%), and Study 3 (*M* = 78.09%). This was not surprising, as Study 4 excluded boys (who tended to perform worse overall on the ANS task). As a result, all subsequent analyses presented in the manuscript control for experiment. Importantly, experiment did not interact with any other variables to predict ANS task performance (*p*s > .11).

#### Gender by beliefs by condition interaction

**Fig 2:**
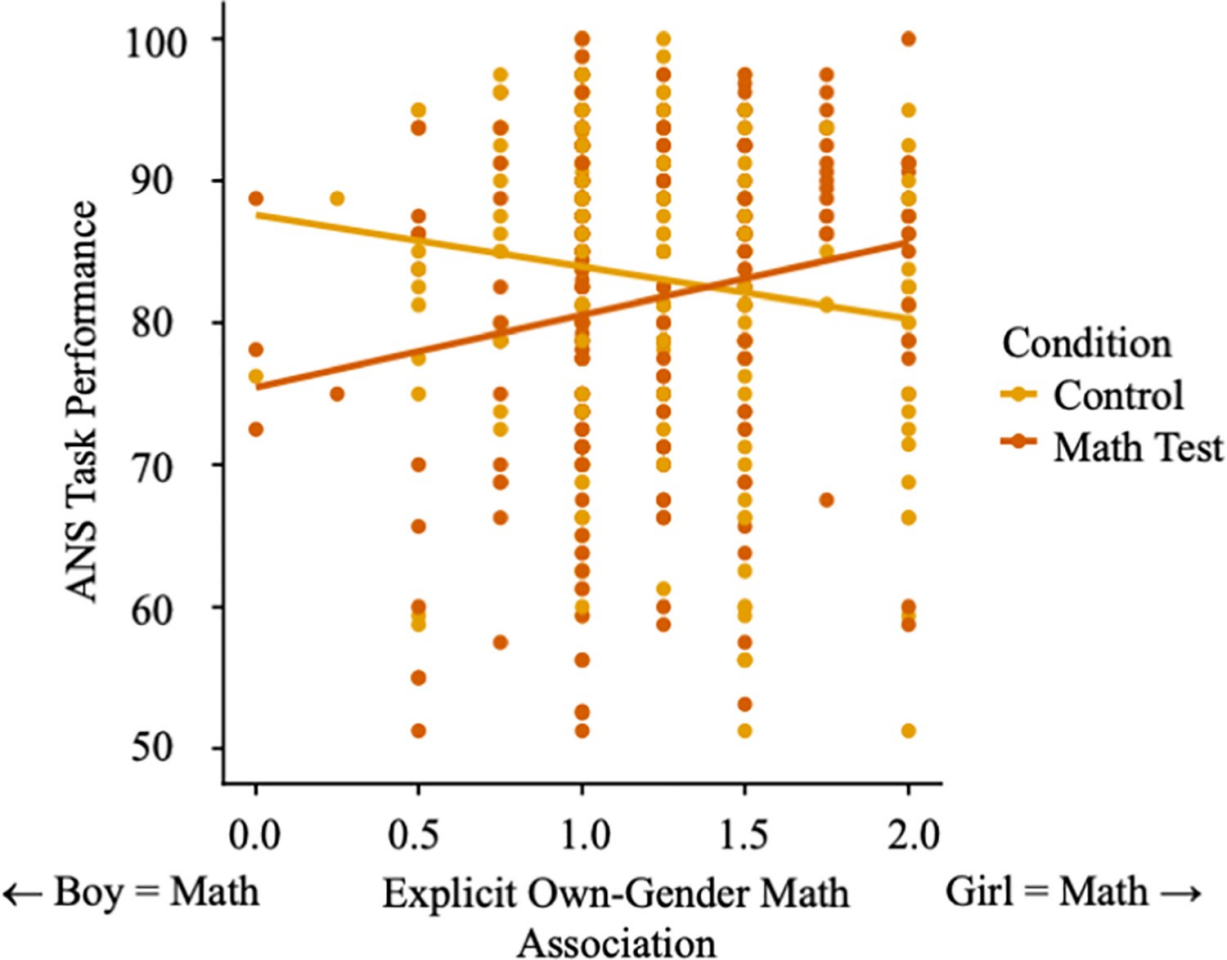
Girls’ ANS task performance by condition.

Analyses on the combined dataset revealed a significant three-way interaction between children’s math own-gender beliefs, child gender, and condition predicting performance on the ANS task, β_int_ = -.32, SE = .14, CI_95_ [-.60, -.04], *p* = .02 (Fig 2). In order to interpret these results, we first examined the significance of each simple two-way interaction for each condition (e.g., an interaction between beliefs and condition predicting ANS accuracy for girls; see S3 Table). For significant two-way interactions, we then conducted simple slope analyses to examine which regression analyses were significant in each subgroup (e.g., whether beliefs predicted ANS accuracy for girls who strongly associated their own gender with math).

##### Beliefs by condition interaction

When examining a potential interaction between math-own gender beliefs and condition, we found that for girls, this interaction was significant, β = .30, SE = .09, CI_95_ [.12, .48], *p* = .001 (see S3 Table). Most notably, simple slopes analyses supported the core hypothesis: girls who associated boys more with math (-1SD from the mean = 0.79) performed worse in the math test condition than the control condition, β = -.42, SE = .13, CI_95_ [-.67, -.17], *p* = .001. This simple effect of condition was non-significant (and reversed in sign) for girls who strongly associated girls with math, +1SD from the mean = 1.63, β = .18, SE = .13, CI_95_ [-.07, .43], *p* = .15. Analyzed differently, in the math test condition, girls who showed a weaker stereotypic association between girls and math tended to exhibit lower ANS task performance, β = .18, SE = .07, CI_95_ [.05, .31], *p* = .006. In the control condition, girls’ math-gender beliefs were not associated with math performance, β = -.12, SE = .07, CI_95_ [-.25, .01], *p* = .07.

**Fig 3:**
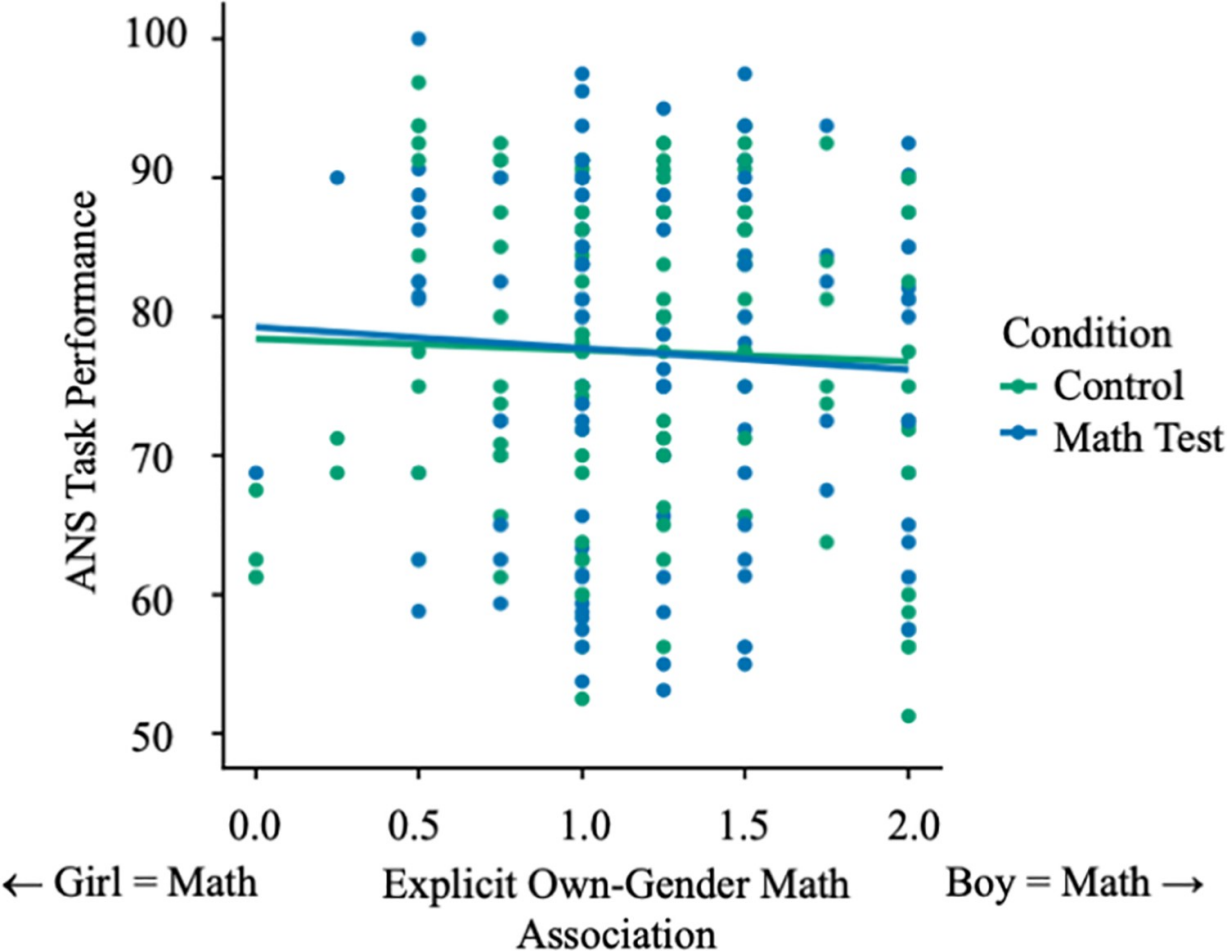
Boys’ ANS task performance by condition.

In contrast to these effects for girls, we found no significant interaction between condition and math-gender beliefs predicting performance on the ANS task for boys, β = -.02, SE = .11, CI_95_ [-.23, .19], *p* = .85 (Fig 3), who performed similarly regardless of condition or beliefs, *M* = 1.23, *SD* = 0.47. In other words, whereas we found an association between gender stereotypes and girls’ intuitive number sense, we did not find this relationship among boys (see Discussion). Further, our manipulation of task description only affected girls’ ANS performance if they had acquired the stereotype associating males more with math, pointing to a potential mechanism underlying this effect.

#### Beliefs by gender interaction

When examining a potential interaction between math-own gender beliefs and child gender in the two different conditions, we found that in the math test condition, this interaction was significant, β = -.23, SE = .10, CI_95_ [-.43, -.03], *p* = .02 (see S3 Table). Simple slopes analyses indicated that for girls in the math test condition, beliefs were a significant predictor of ANS performance; girls with a weaker association between girls and math tended to exhibit lower ANS task performance, β = .18, SE = .07, CI_95_ [.05, .31], *p* = .006. This was not the case for boys in the math test condition, β = -.05, SE = .08, CI_95_ [-.21, .10], *p* = .51. Analyzed as simple gender effects, we found that for children who strongly associated their own gender with math, girls performed significantly better than boys, β = -.47, SE = .15, CI_95_ [-.77, -.18], *p* = .002. This gender difference was not significant for children who did not strongly associate their own gender with math, β = -.01, SE = .15, CI_95_ [-.30, .29], *p* = .96.

In the control condition, we found no significant interaction between gender and math-gender beliefs predicting performance on the ANS task, β = .09, SE = .10, CI_95_ [-.10, .28], *p* = .37. Boys and girls performed similarly regardless of beliefs, *M* = 80.08, *SD* = 11.62. Overall, we found an association between math-own gender beliefs and ANS task performance for girls in the math test condition, but not for boys. Furthermore, while girls who associated their own gender with math outperformed boys who associated their own gender with math, this gender difference in performance disappeared in the math test condition.

#### Condition by gender interaction

When examining a potential interaction between condition and child gender predicting ANS performance, we found that when children had a weak (or reversed) association between their own gender and math (-1 SD from the mean = 0.78), this interaction was significant, β = .42, SE = .20, CI_95_ [.02, .82], *p* = .04 (see S3 Table). As described above, simple slopes analyses indicated that girls who associated math more with boys performed worse in the math test condition than the control condition, β = -.42, SE = .13, CI_95_ [-.67, -.17], *p* = .001. This simple effect of condition was not significant among those boys who associated math more with girls, β = .004, SE = .16, CI_95_ [-.31, .32], *p* = .98.

Analyzed differently, for children in the control condition with a weak association between their own gender and math, or an association between the other gender and math, girls performed significantly better than boys, β = -.43, SE = .15, CI_95_ [-.72, -.14], *p* = .004. Gender did not predict ANS performance for children in the math test condition who did not strongly associate their own gender with math, β = -.01, SE = .15, CI_95_ [-.30, .29], *p* = .96.

When children had a strong association between their own gender and math, there was no significant interaction between condition and child gender predicting performance on the ANS task, β = -.22, SE = .20, CI_95_ [-.18, .62], *p* = .28. Performance was comparable for boys and girls who strongly associated their own gender with math across conditions, *M* = 80.47, *SD* = 11.30. In summary, we found that specifically girls who associated math more with boys did worse when told the ANS task was a test. In contrast, these girls outperformed boys when they were told the task was a game or an eyesight test.

#### Individual study results

The analyses summarized above represent the highest power test of our hypothesis. Nonetheless, there is some descriptive variation across the studies summarized here (see also S4 and S5 Tables). See Supporting Information for a description of results broken down by individual study (Data in S1–S4 Texts).

#### Math-gender beliefs

Across all samples children had a significant tendency (except for girls in study 1, *p* = .11) to associate their own gender with math. Furthermore, in all studies, these beliefs did not differ significantly by condition. Importantly, we never observed evidence in any study that girls (3–6 year olds) on average believed boys to be significantly better than girls at math and counting. In fact, our decision to analyze a combined dataset emerged over time (and was preregistered prior to Study 4) as it became clear that each individual study revealed only a small proportion of girls who held more stereotypic beliefs.

#### ANS task performance

Across the three studies that include both boys and girls (Study 1–3), the three-way interaction yielded a similar negative coefficient (β‘s = -.59, -.37, -.44), although this interaction is only significant in the highly powered mega-analysis (see S3 and S4 Tables). The more focused stereotype beliefs x condition interaction among girls was only significant in Study 2, though trending in the same direction in Study 1 and 3 (but not 4). This same interaction was never present or even trending, *p*s > .71, among boys. Simple slopes analyses also revealed that describing the task as a math test (as opposed to game or eye test) led to significantly lower ANS performance only among girls with negative stereotype beliefs in Studies 2 and 3 (not Studies 1 and 4), although this effect was significant in the mega-analysis. This simple slope was never significant among girls who do not hold this stereotype or among boys regardless of their beliefs.

## Discussion

Overall, gender-math beliefs were indicative of an in-group preference in our sample; 3–6 year old boys and girls, on average, endorsed a belief that their own gender was better at math. This finding complements existing research suggesting that young children display in-group favoritism in regard to math-gender beliefs [11]. Importantly, we also show there is clear variability in this association. Among girls across our sample who endorsed gender stereotypes about girls’ lower math ability (n = 60), framing a task as a math and counting test affected their performance on a basic and universal assessment of number intuition. Although girls generally performed better than boys on the ANS task, this gender difference was eliminated when the task was described as a math test and for those girls who believe that boys are stereotypically better at math. Thus, girls’ beliefs about math are related to their intuitive number sense specifically when these stereotypes are activated in a testing context. As such, it is possible that both stereotype endorsement and activation through contextual cues must be present for stereotypes about gender and math to affect young girls’ math ability in early childhood. Such early impairments could set the stage for larger gender gaps in math performance and interest if these effects also shape girls’ and boys’ emerging attitudes and self-confidence in one’s math abilities, or their actual ability to learn formal math concepts that are supported by ANS.

We also found an overall gender difference in ANS performance, with girls outperforming boys when stereotypes were not activated. These results are consistent with evidence that school-aged girls often outperform boys in math, albeit by a smaller margin than language arts [43]. Our evidence for girls’ excelling at a test of their math performance in early development even further highlights the importance of understanding how cultural beliefs like stereotypes may draw girls away from math-intensive fields over time. However, we note that our findings stand in contrast to other meta-analytic work suggesting that there are no gender differences in children’s math performance, and in particular, no gender differences in ANS acuity [32, 44]. It is possible that this difference may have been driven by the atypical instructions presented in this task, or by the unique testing environment (a community science center). Future work should seek to replicate and explore the causes of the gender difference we found, as well as examine whether or not young girls’ comparable performance in math relative to boys might actually be underperformance in respect to their potential [45].

While these results indicate that preschool girls’ number sense can be impacted by stereotype activation, boys in our study were unaffected. On the one hand, the lack of effects for boys is somewhat surprising given that if a certain percentage (19% of boys in our sample) hold the belief that girls should be better at math and counting, one might expect their performance to be impaired when they think they are being tested in this domain [22]. However, there are other similar cases of gender asymmetries, where girls show greater sensitivities to gender stereotypes than do boys. For example, young girls appear to internalize their parents’ gender biases more than young boys do [46]. Moreover, other work shows that boys are slower to internalize stereotypes about math and gender [19, 20]. In line with this evidence, we speculate that boys in our study may have been less sensitive to gender stereotype activation.

At a surface level, the pattern of results in this study appear comparable to stereotype threat effects that have been found with older girls and adult women (e.g. [5, 8, 11, 12]). However, the mechanisms behind these effects in adult samples are most likely different for young girls. In women, stereotype threat effects are proposed to stem from anxiety about confirming stereotypes about one’s own group and self-conscious performance monitoring which can hijack the same working memory resources needed for complex mathematical performance [47]. In contrast, for young girls, it seems more likely that those who have stereotypes about math and gender may simply disengage from the task at hand when these stereotypes are activated, which would be a different mechanism than stereotype threat. Future research should examine whether these stereotype-based performance effects are similar to stereotype threat.

One limitation to the present set of studies is that our ability to detect these effects in individual studies is hampered by the fact that very few girls aged 3–6 hold the explicit belief that boys are better than girls at math and counting. In our combined dataset of 498 girls, only 60 girls held this typical math-gender stereotype (quantified as a math-gender stereotype score below 1). It was for this small subsample that framing the task as a test of math ability significantly lowered their ANS performance compared to the control condition, β = .42, *p* < .001. Descriptively, this low number of girls who hold the stereotype could be a promising sign. It might reflect the fact that gender stereotypes about intelligence have been favoring girls over time [16, 17] or that children at this young age have not yet been exposed to stereotypic beliefs about girls and math. Given that children were recruited at a science center, the sample might also overrepresent children, and especially girls, whose parents already hold or try to actively counter gender stereotypes about math and science. From a statistical power standpoint, the fact such a small percentage of girls hold the stereotype means that effects are difficult to detect in typically sized samples.

Given the low baserates of stereotype beliefs, future research examining stereotype threat among children will need to be sensitive to variation in children’s knowledge and beliefs about gender stereotypes. Previous research has found mixed results of the effect of stereotype activation and stereotype threat on children’s math test performance [13, 14]. The present results add to other evidence suggesting that one factor could be variability in the stereotype knowledge and beliefs that children have [21, 22]. Furthermore, in this age group, it is not uncommon for young children to display in-group favoritism in their explicit responses [48]. This in-group bias was present within our data and likely competes against the cultural stereotype, even if children have been exposed to those stereotypic beliefs and associations. Despite these countervailing effects of ingroup biases, the individual variability in beliefs predicted girls’ susceptibility to stereotype effects. Future studies should ensure measurement of children’s stereotypes as key moderators of the effect of contextual cues on math performance.

Though only a handful of girls were impacted by our stereotype framing, these particular girls may be at risk for reduced performance in mathematics domains when they enter a formal schooling environment. It should be noted that the size of this group does not diminish the importance of addressing these stereotypes early in development, as this effect has the potential to create long-lasting inequity among young girls. In conjunction with past work, these results suggest that even though both genders start off on a level playing field in terms of foundational math abilities, activation of internalized math-gender stereotypes may begin to tip the scales quite early in development for some young girls by decreasing their ANS accuracy–just as this ability could aid them in learning formal mathematical concepts. If contextual activation of stereotypes can impair the basic numerical abilities of preschool girls who endorse stereotypes about gender and math, these effects might compound across development to prevent girls from achievement in mathematics [49]. Thus, interventions to increase girls’ engagement in math and math-related fields should consider starting very early in development, before gender stereotypes can create a cycle of impaired performance and reduced interest in math.

## Supporting information

S1 Fig Flowchart of differences between Study 1–4. (TIFF)

S1 Table Participant exclusions by study. (PDF)

S2 Table Means and standard deviations for math-gender beliefs and ANS accuracy. (PDF)

S3 Table Table of coefficients from decomposed interactions predicting ANS accuracy by individual study. (PDF)

S4 Table Means and standard deviations for math-gender beliefs and ANS accuracy by individual study. (PDF)

S5 Table Table of coefficients from decomposed interactions predicting ANS accuracy by individual study. (PDF)

S1 Text Study 1 results. (PDF)

S2 Text Study 2 results. (PDF)

S3 Text Study 3 results. (PDF)

S4 Text Study 4 results. (PDF)
