# Supplementary figures and images for: The effect of gender stereotypes on young girls’ intuitive number sense
Source: PLoS One. 2021 Oct 28;16(10):e0258886. doi: 10.1371/journal.pone.0258886 (PMC8553059; doi:10.1371/journal.pone.0258886)

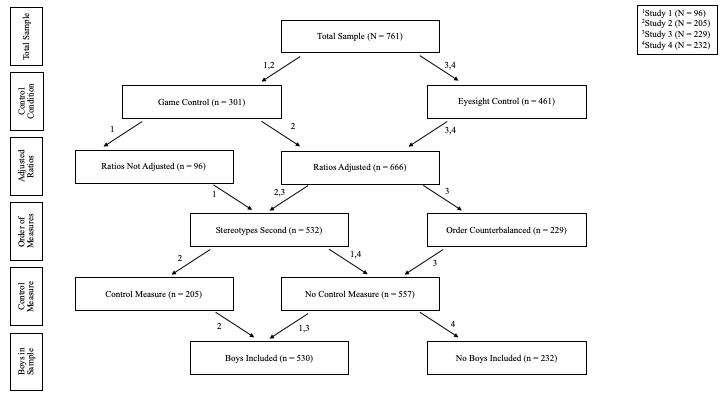

Supplement: S1 Fig — (TIFF) [file pone.0258886.s001.tiff]
