# Supplementary material for: The effect of gender stereotypes on young girls’ intuitive number sense
Source: PLoS One. 2021 Oct 28;16(10):e0258886. doi: 10.1371/journal.pone.0258886 (PMC8553059; doi:10.1371/journal.pone.0258886)
Supplement: S1 Table — (PDF) [file pone.0258886.s002.pdf]

S1 Table. Participant exclusions by study.

Exclusion	Study 1	Study 2	Study 3	Study 4	Total
Random pressing	19	29	20	7	75
Did not finish	13	42	47	32	134
Family interference	1	5	1	3	10
Language barrier	5	8	8	9	30
RA/Tech error	10	2	1	7	20
Neurodivergent	0	0	2	0	2
<50% correct	3	1	6	2	12
Total	51	87	85	60	283
