# Supplementary material for: The effect of gender stereotypes on young girls’ intuitive number sense
Source: PLoS One. 2021 Oct 28;16(10):e0258886. doi: 10.1371/journal.pone.0258886 (PMC8553059; doi:10.1371/journal.pone.0258886)
Supplement: S2 Table — (PDF) [file pone.0258886.s003.pdf]

S2 Table. Means and standard deviations for math-gender beliefs and ANS accuracy.

	Mean	Standard Deviation
Math-Own Gender Beliefs[a]	1.20	0.42
Girls	1.20	0.39
Boys	1.20	0.47
Math	1.20	0.41
Control	1.21	0.43
Girls/Math	1.18	0.39
Girls/Control	1.23	0.40
Boys/Math	1.22	0.45
Boys/Control	1.18	0.49
ANS Accuracy[b]	80.61	11.41
Girls	82.29	10.93
Boys	77.44	11.63
Math	81.15	11.18
Control	80.08	11.62
Girls/Math	81.49	11.23
Girls/Control	83.11	10.58
Boys/Math	77.40	11.93
Boys/Control	77.47	11.38

[a]Range from 0 to 2, with higher numbers indicating a stronger association between one's own gender and math.
[b]ANS performance is a percentage of correct trials.
