# Supplementary material for: The effect of gender stereotypes on young girls’ intuitive number sense
Source: PLoS One. 2021 Oct 28;16(10):e0258886. doi: 10.1371/journal.pone.0258886 (PMC8553059; doi:10.1371/journal.pone.0258886)
Supplement: S3 Table — (PDF) [file pone.0258886.s004.pdf]

S3 Table. Table of coefficients from decomposed interactions predicting ANS accuracy.

Predictor<br>Condition	β	*SE*	*t*	*p*
Beliefs x Condition				
Girls	.30	.09	3.23	.001
Boys	-.02	.11	0.20	.85
Beliefs x Gender				
Control	.09	.10	0.91	.37
Math	-.23	.10	2.28	.023
Condition x Gender				
Low Own-Gender=Math	.42	.20	2.07	.039
High Own Gender=Math	-.22	.20	1.08	.28
Condition[a]				
Girls/Low	-.42	.13	3.28	.001
Girls/High	.18	.13	1.44	.15
Boys/Low	.004	.16	0.03	.98
Boys/High	-.04	.16	0.24	.82
Beliefs				
Math/Girls	.18	.07	2.75	.006
Math/Boys	-.05	.08	0.66	.51
Control/Girls	-.12	.07	1.82	.070
Control/Boys	-.03	.07	0.42	.67
Gender[b]				
Math/Low	-.01	.15	0.06	.96
Math/High	-.47	.15	3.16	.002
Control/Low	-.43	.15	2.89	.004
Control/High	-.25	.15	1.70	.090

[a]Condition coded as 0 = control, 1 = math test.
[b]Gender coded as 0 = F, 1 = M.
