# Supplementary material for: The effect of gender stereotypes on young girls’ intuitive number sense
Source: PLoS One. 2021 Oct 28;16(10):e0258886. doi: 10.1371/journal.pone.0258886 (PMC8553059; doi:10.1371/journal.pone.0258886)
Supplement: S4 Table — (PDF) [file pone.0258886.s005.pdf]

S4 Table. Means and standard deviations for math-gender beliefs and ANS accuracy by individual study.

	Study 1	Study 2	Study 3	Study 4
Math-Gender Beliefs	1.15 (.52)	1.17 (.40)	1.25 (.43)	N/A
Girls	1.19 (.54)	1.16 (.37)	1.24 (.36)	1.21 (.39)
Boys	1.11 (.50)	1.18 (.44)	1.27 (.49)	N/A
Math	1.13 (.51)	1.19 (.40)	1.24 (.40)	1.20 (.40)
Control	1.16 (.53)	1.15 (.40)	1.27 (.46)	1.22 (.38)
Girls/Math	1.12 (.57)	1.19 (.35)	1.19 (.32)	1.20 (.40)
Girls/Control	1.31 (.50)	1.13 (.39)	1.30 (.39)	1.22 (.38)
Boys/Math	1.15 (.45)	1.19 (.45)	1.29 (.46)	N/A
Boys/Control	1.08 (.54)	1.17 (.42)	1.24 (.52)	N/A
ANS Accuracy	77.10 (12.34)	81.12 (11.02)	78.09 (12.21)	84.09 (9.39)
Girls	79.05 (14.07)	81.80 (10.73)	80.34 (12.12)	84.09 (9.39)
Boys	75.58 (10.68)	80.39 (11.34)	75.71 (11.91)	N/A
Math	76.92 (12.23)	80.84 (11.43)	77.10 (12.51)	83.92 (9.22)
Control	77.29 (12.58)	81.41 (10.62)	79.15 (11.86)	84.25 (9.58)
Girls/Math	79.16 (13.33)	81.09 (10.74)	78.43 (13.12)	83.92 (9.22)
Girls/Control	78.87 (15.66)	82.50 (10.76)	82.46 (10.63)	84.25 (9.58)
Boys/Math	74.49 (10.66)	80.59 (12.17)	75.63 (11.73)	N/A
Boys/Control	76.46 (10.80)	80.15 (10.43)	75.78 (12.19)	N/A
