# Supplementary material for: The effect of gender stereotypes on young girls’ intuitive number sense
Source: PLoS One. 2021 Oct 28;16(10):e0258886. doi: 10.1371/journal.pone.0258886 (PMC8553059; doi:10.1371/journal.pone.0258886)
Supplement: S5 Table — (PDF) [file pone.0258886.s006.pdf]

S5 Table. Table of coefficients from decomposed interactions predicting ANS accuracy by individual study.

Predictor	Study 1			Study 2			Study 3			Study 4[a]		
Condition	β(*SE*)	*t*	*p*	β(*SE*)	*t*	*p*	β(*SE*)	*t*	*p*	β(*SE*)	*t*	*p*
Beliefs x Condition x Gender	-.59 (.44)	1.34	.18	-.37 (.28)	1.31	.19	-.44 (.27)	1.61	.11	-	-	-
Beliefs x Condition												
Girls	.57 (.32)	1.75	.19	.46 (.21)	2.18	.031	.38 (.22)	1.74	.083	-.12 (.19)	0.63	.53
Boys	-.02 (.30)	0.08	.94	.09 (.19)	0.50	.62	-.06 (.17)	0.37	.71	-	-	-
Beliefs x Gender												
Control	.25 (.32)	0.77	.44	-.06 (.20)	0.32	.75	.11 (.18)	0.61	.54	-	-	-
Math	-.34 (.30)	1.13	.26	-.44 (.20)	2.16	.032	-.33 (.21)	1.61	.11	-	-	-
Condition x Gender												
Low Beliefs	.43 (.64)	0.67	.51	.55 (.40)	1.38	.17	.74 (.38)	1.96	.051	-	-	-
High Beliefs	-.75 (.58)	1.29	.20	-.19 (.40)	0.48	.63	-.15 (.38)	0.38	.70	-	-	-
Condition[b]												
Girls/Low	-.56 (.51)	1.14	.26	-.60 (.29)	2.11	.037	-.68 (.28)	2.45	.015	.02 (.27)	0.07	.94
Girls/High	.56 (.41)	1.36	.18	.33 (.29)	1.12	.26	.08 (.29)	0.27	.79	.26 (.27)	0.96	.34
Boys/ Low	-.15 (.40)	0.37	.71	-.05 (.28)	0.18	.86	.06 (.25)	0.22	.83	-	-	-
Boys/High	-.19 (.41)	0.46	.64	.14 (.27)	0.50	.62	-.07 (.25)	0.27	.79	-	-	-
Beliefs												
Math/Girls	.40 (.18)	2.19	.031	.37 (.16)	2.36	.020	.26 (.16)	1.58	.12	-.11 (.14)	0.83	.41
Math/Boys	.06 (.24)	0.24	.81	-.06 (.12)	0.49	.62	-.07 (.12)	0.57	.57	-	-	-

Control/Girls	-.17 (.27)	0.63	.53	-.09 (.14)	0.63	.53	-.12 (.14)	0.83	.41	.01 (.14)	0.06	.95
Control/Boys	.08 (.18)	0.45	.65	-.15 (.14)	1.08	.28	-.01 (.11)	0.09	.93	-	-	-
Gender[c]												
Math/Low	-.06 (.41)	0.15	.88	.41 (.29)	1.44	.15	.07 (.27)	0.26	.79	-	-	-
Math/High	-.74 (.42)	1.78	.078	-.46 (.27)	1.69	.093	-.59 (.29)	2.08	.039	-	-	-
Control/Low	-.49 (.49)	0.99	.33	-.14 (.28)	0.50	.62	-.12 (.14)	0.83	.013	-	-	-
Control/High	.01 (.41)	0.02	.98	-.27 (.29)	0.92	.36	-.01 (.11)	0.09	.076	-	-	-

[a]Study 4 only included female participants
[b]Condition coded as 0 = control, 1 = test
[c]Gender coded as 0 = F, 1 = M
