# Supplementary material for: The effect of gender stereotypes on young girls’ intuitive number sense
Source: PLoS One. 2021 Oct 28;16(10):e0258886. doi: 10.1371/journal.pone.0258886 (PMC8553059; doi:10.1371/journal.pone.0258886)
Supplement: S1 Text — (PDF) [file pone.0258886.s007.pdf]

Study 1 Results

Math-gender beliefs

Our first study acted as a pilot study to examine potential effects of task framing on children's ANS performance by gender. We found no gender differences in the magnitude of math-gender beliefs (see Table S2), as boys and girls had comparable associations between their own gender and math, $t(94) = -.74$, $p = .46$, $d = 0.15$. Boys significantly explicitly associated their own gender with math, boys: $t(41) = 2.27$, $p = .028$, and girls had a marginally significant explicit association between their own gender and math $t(53) = 1.64$, $p = .11$. Furthermore, there were no differences in the magnitude of beliefs across conditions, as mean levels of math-gender beliefs were comparable across the game, $M = 1.16$, $SD = .53$, and math test, $M = 1.13$, $SD = .51$, conditions, $t(94) = .31$, $p = .76$, $d = 0.06$. As defined by greater than one standard deviation below the mean (Belief Score <= 0.79), a total of 11 girls in this study were considered to have a strong association between boys and math.

ANS task performance

In a similar manner as our key regression analyses, we entered math-gender beliefs, child gender, and condition as predictors of ANS task performance (see Table S3). The three-way interaction between children's math-gender beliefs, child gender, and condition was non-significant, most likely as a result of our study being very underpowered to detect the interaction, $\beta_{int} = -.59$, CI_{95} [-1.30, .25], $p = .184$. When conducting exploratory analyses to examine the performance of girls in the Math Test condition, we found that girls' math-gender beliefs significantly predicted their ANS performance, $\beta_{int} = .40$, CI_{95} [.03, .67], $p = .031$. Based on this finding, we chose to pursue additional follow-up studies with more power.
