# Supplementary material for: The effect of gender stereotypes on young girls’ intuitive number sense
Source: PLoS One. 2021 Oct 28;16(10):e0258886. doi: 10.1371/journal.pone.0258886 (PMC8553059; doi:10.1371/journal.pone.0258886)
Supplement: S2 Text — (PDF) [file pone.0258886.s008.pdf]

Study 2 Results

Math-gender beliefs

We compared children's explicit math-gender beliefs by gender and condition (see Table S2). There was no gender difference in the magnitude of math-gender beliefs, as boys and girls had comparable associations between their own gender and math, $t(227) = -.46$, $p = .65$, $d = -0.06$. Both boys and girls on average explicitly associated their own gender with math, boys: $t(98) = 4.09$, $p < .001$; girls: $t(105) = 4.39$, $p < .001$. Furthermore, there were no differences in the magnitude of beliefs across conditions, as mean levels of math-gender beliefs were comparable across the game, $M = 1.16$, $SD = .40$, and math test, $M = 1.19$, $SD = .40$, conditions, $t(227) = .62$, $p = .54$, $d = 0.08$. As defined by greater than one standard deviation below the mean (Belief Score <= 0.79), a total of 17 girls in this study were considered to have a strong association between boys and math.

ANS task performance

We entered math-gender beliefs, child gender, and condition as predictors of ANS task performance (see Table S3). The three-way interaction between children's math-gender beliefs, child gender, and condition was non-significant, potentially as a result of our study being underpowered, $\beta_{int} = -.37$, CI_{95} [-.93, .19], $p = .193$. However, for girls, there was a significant interaction between math-gender beliefs and condition, $\beta = .46$, CI_{95} [.04, .88], $p = .031$. Girls who associated boys with math performed worse in the math test condition than the game condition, $\beta = -.60$, CI_{95} [-1.16, -.04], $p = .037$. This effect was non-significant for girls who strongly associated girls with math, $\beta = .33$, CI_{95} [-.25, .90], $p = .263$. Thus, it appears that girls

who endorsed math-gender stereotypes associating boys more with math had worse performance when these stereotypes were activated.

Analyzed differently, in the Math Test condition, girls' beliefs about gender and math predicted their ANS performance; girls with a weaker association between girls and math performed worse on the ANS task than girls with a stronger association, $\beta = .37$, CI_{95} [.06, .69], $p = .020$. These results suggest that when math-gender stereotypes were activated, they predicted girls' ANS task performance. This was not the case in the game condition, where girls' math-gender beliefs were not significantly associated with ANS performance, $\beta = -.09$, CI_{95} [-.37, .19], $p = .530$. We found no significant interaction between condition and math-gender beliefs predicting performance on the ANS task for boys, who performed similarly regardless of condition or beliefs, $\beta = .09$, CI_{95} [-.28, .46], $p = .621$.

In addition to these analyses, we once again looked at lower-order interactions with gender as a moderator. In the math test condition, there was a significant interaction between children's math-gender beliefs and their gender predicting ANS performance, $\beta = -.44$, CI_{95} [-.83, -.04], $p = .032$. There was no interaction between math-gender beliefs and child gender in the control condition, $\beta = -.06$, CI_{95} [-.46, .33], $p = .750$. Simple slopes analyses indicated that gender did not significantly predict ANS task performance regardless of condition or beliefs (*p*s $> .093$), suggesting that these interactions were not driven by gender differences in performance.

Control measure

We included a control measure to ensure that children were not simply selecting their own gender regardless of question content. To test whether or not children had an overall gender

response bias, we examined whether there was a correlation between children's beliefs about math and gender, and their beliefs about a novel word ("daxing") and gender.

The score for children's beliefs about daxing and gender was calculated by coding the beliefs in a similar manner to the math-gender beliefs, according to participants' own gender (0 = other gender – daxing association, 2 = own gender – daxing association). The questions about daxing interest and ability were then averaged to create an overall daxing-gender association score ($M = 1.1$, $SD = .56$). When tested against chance (midpoint = 1), a one-sample t-test indicated that children significantly associated daxing with their own gender, $t(204) = 27.86$, $p < .001$. However, we observed no correlation between children's math-gender association and their daxing-gender association, $r(203) = .02$, $p = .836$, suggesting that children were not simply answering the questions about math and gender based on an overall gender preference. Furthermore, there was no correlation between daxing-gender beliefs and ANS task performance, $r(203) = -.13$, $p = .073$.
