# Supplementary material for: The effect of gender stereotypes on young girls’ intuitive number sense
Source: PLoS One. 2021 Oct 28;16(10):e0258886. doi: 10.1371/journal.pone.0258886 (PMC8553059; doi:10.1371/journal.pone.0258886)
Supplement: S3 Text — (PDF) [file pone.0258886.s009.pdf]

Study 3 Results

Math-gender beliefs

For Study 3, we again compared children's explicit math-gender beliefs by gender and condition (see Table S2). Boys, $M = 1.26$, $SD = .48$, and girls, $M = 1.24$, $SD = .36$, had comparable associations between their own gender and math, $t(246) = .33$, $p = .74$, $d = 0.05$. Both boys and girls on average explicitly associated their own gender with math, boys: $t(110) = 5.73$, $p < .001$, girls: $t(117) = 7.19$, $p < .001$). Similarly, math-gender beliefs were not significantly different between the eyesight, $M = 1.26$, $SD = .46$, and math test, $M = 1.24$, $SD = .39$, conditions, $t(246) = .41$, $p = .68$, $d = 0.05$. As defined by greater than one standard deviation below the mean (Belief Score <= 0.79), a total of 9 girls in this study were considered to have a strong association between boys and math.

ANS task performance

Analyses were conducted in the same way as previous studies (see Table S3). The three-way interaction between children's math-gender beliefs, child gender, and condition was non-significant, $\beta_{int} = -.44$, CI_{95} [-.98, .10], $p = .108$. For girls, there was a marginally significant interaction between math-gender beliefs and condition, $\beta = .38$, CI_{95} [-.05, .81], $p = .086$. However, as our effect size was similar to Study 2, we explored the simple slopes of this interaction to see if results were consistent. Girls who associated boys with math performed worse in the Math Test condition than the Eyesight condition, $\beta = -.68$, CI_{95} [-1.23, -.13], $p =$

.015. This effect was non-significant for girls who strongly associated girls with math, $\beta = .08$, CI_{95} [-.50, .66], $p = .827$.

In the math test condition, girls' beliefs about gender and math did not significantly predict their ANS performance, though effect sizes were consistent with previous studies, $\beta = .26$, CI_{95} [-.07, .59], $p = .116$. In the eyesight condition, girls' math-gender beliefs were also not significantly associated with ANS performance, $\beta = -.12$, CI_{95} [-.40, .16], $p = .405$. We found no significant interaction between condition and math-gender beliefs predicting performance on the ANS task for boys, who performed similarly regardless of condition or beliefs, $\beta = -.06$, CI_{95} [-.39, .26], $p = .712$.

Once again, we looked at lower-order interactions with gender as a moderator in order to examine potential gender differences. In both the math test condition, $\beta = -.33$, CI_{95} [-.74, .07], $p = .109$, and the control condition, $\beta = .11$, CI_{95} [-.25, .47], $p = .544$, the interaction between children's math-gender beliefs and their gender was non-significant.

Order analyses

One of our concerns after Study 1 and 2 was that children might respond to our measure of explicit stereotypes based on their own performance (e.g. if the child did well, they might say that their gender does better based on that self-assessment). To address this issue in Study 3, we counterbalanced the order in which the explicit questions were presented. To ensure that results were robust controlling for potential order effects, we also ran the regression analyses controlling for order. Order was not found to be a significant covariate, $\beta = -.12$, CI_{95} [-.38, .14], $p = .355$, and overall results were nearly identical.

As an additional exploratory analysis, we added order as a moderator to look at a potential three-way interaction between gender, condition and order. Consistent with the

aforementioned analyses, there was a main effect of gender, $F(1,221) = 9.04$, $p = .003$, $\eta p^2 = .04$, such that girls performed better on the ANS task than boys. There were no main effects of condition, $F(1,221) = 1.36$, $p = .245$, $np^2 = .006$ or order, $F(1,221) = 1.17$, $p = .281$, $\eta p^2 = .005$ predicting ANS task performance. Additionally, there were no two-way interactions between gender, condition, and order (*p*s > .203). However, there was a three-way interaction between gender, condition, and order predicting ANS performance, $F(1,221) = 7.51$, $p = .007$, $\eta p^2 = .033$. In order to analyze this interaction, we performed analyses for each order separately. We did not conduct regression analyses of variation in stereotype belief for the samples split by order due to inadequate power for these higher order interactions.

Explicit questions before ANS task

Our first analysis examined if girls' ANS accuracy was negatively impacted by stereotypes about boys being better at math when asked about these stereotypes before the task. Consistent with the overall findings of the study, we found a marginally significant main effect of gender, $F(1,111) = 3.74$, $p = .056$, $\eta p^2 = .03$, such that girls performed better than boys. There was no main effect of condition, $F(1,111) = .42$, $p = .521$, $\eta p^2 = .004$ but there was a gender by condition interaction predicting ANS task performance, $F(1,111) = 9.50$, $p = .003$, $\eta p^2 = .08$. Simple effects analyses indicated that girls performed significantly worse in the math test condition, $M = 77.18$, $SD = 12.86$, as compared to the eyesight condition, $M = 84.82$, $SD = 7.18$; $p = .007$. Thus, asking girls about their math-gender stereotypes before the ANS task may have served to strengthen the manipulation, resulting in an effect of stereotypes on performance regardless of explicit stereotype endorsement.

Explicit questions after ANS task

Next, we examined whether girls' ANS acuity was negatively impacted when questions about gender stereotypes came after the task (as they had been placed in Study 1 and 2). We again found a main effect of gender, $F(1,110) = 5.30$, $p = .023$, $\eta p^2 = .05$, such that girls performed better than boys. There was no main effect of condition, $F(1,110) = .96$, $p = .328$, $\eta p^2 = .009$, and no gender by condition interaction, $F(1,110) = .93$, $p = .337$, $\eta p^2 = .008$. These results indicate when girls were asked about their beliefs after the ANS task, there was no general effect of priming stereotypes on girls' ANS performance.
