# Supplementary material for: The effect of gender stereotypes on young girls’ intuitive number sense
Source: PLoS One. 2021 Oct 28;16(10):e0258886. doi: 10.1371/journal.pone.0258886 (PMC8553059; doi:10.1371/journal.pone.0258886)
Supplement: S4 Text — (PDF) [file pone.0258886.s010.pdf]

Study 4 Results

Math-gender beliefs

Girls on average explicitly associated their own gender with math (see Table 3.2), $t(231) = 8.23$, $p < .001$. There were no differences in the magnitude of beliefs across conditions, as mean levels of math-gender beliefs were comparable across the eyesight, $M = 1.22$, $SD = .38$, and math test, $M = 1.20$, $SD = .40$ conditions, $t(230) = -.47$, $p = .64$, $d = 0.05$. As defined by greater than one standard deviation below the mean (Belief Score <= 0.79), a total of 23 girls in this study were considered to have a strong association between boys and math.

ANS task performance

In a similar manner as our key regression analyses, we entered math-gender beliefs, child gender, and condition as predictors of ANS task performance (see Table 3). The interaction between math-gender beliefs and condition was not significant, $\beta = -.12$, CI_{95} [-.08, .38], $p = .53$. As the main effect of interest was significant in our previous studies, but not in this study, $\beta = -.11$, CI_{95} [-.17, .16], $p = .41$, we decided to perform a mega-analysis on the combined dataset to examine this small, but potentially impactful effect of the Math Test condition on the ANS performance of girls who associated math with boys.
